# Different sensitivity and threshold in response to nitrogen addition in four alpine grasslands along a precipitation transect on the Northern Tibetan Plateau

**DOI:** 10.1002/ece3.5514

**Published:** 2019-08-01

**Authors:** Ning Zong, Guangshuai Zhao, Peili Shi

**Affiliations:** ^1^ Lhasa National Ecological Research Station, Key Laboratory of Ecosystem Network Observation and Modelling, Institute of Geographic Sciences and Natural Resources Research Chinese Academy of Sciences Beijing China; ^2^ China National Forestry Economics and Development Research Center Beijing China; ^3^ University of Chinese Academy of Sciences Beijing China

**Keywords:** alpine grasslands, nitrogen addition, precipitation gradient, saturation threshold, sensitivity, The Changtang Plateau

## Abstract

The increase in atmospheric nitrogen (N) deposition has resulted in some terrestrial ecological changes. In order to identify the response of sensitive indicators to N input and estimate the sensitivity and saturation thresholds in alpine grasslands, we set up a series of multilevel N addition experiments in four types of alpine grasslands (alpine meadow [AM], alpine meadow‐steppe [AMS], alpine steppe [AS], and alpine desert‐steppe [ADS]) along with a decreasing precipitation gradient from east to west on the Northern Tibetan Plateau. N addition only had significant effects on species diversity in AMS, while had no effects on the other three alpine grasslands. Aboveground biomass of grasses and overall community in ADS were enhanced with increasing N addition, but such effects did not occur in AS. Legume biomass in ADS and AS showed similar unimodal patterns and exhibited a decreasing tend in AM. Regression fitting showed that the most sensitive functional groups were grasses, and the N saturation thresholds were 103, 115, 136, and 156 kg N hm^−2^ year^−1^ in AM, AMS, AS, and ADS, respectively. This suggests that alpine grasslands become more and more insensitive to N input with precipitation decrease. N saturation thresholds also negatively correlated with soil N availability. N sensitivity differences caused by precipitation and nutrient availability suggest that alpine grasslands along the precipitation gradient will respond differently to atmospheric N deposition in the future global change scenario. This different sensitivity should also be taken into consideration when using N fertilization to restore degraded grasslands.

## 1 INTRODUCTION

In the last century, human activities have resulted in more than threefold increase in atmospheric nitrogen (N) deposition all over the world (Galloway et al., 2004, 2008; Lamarque et al., 2005). The increase in N deposition has reached a level that dramatically influencing the stability and function of some natural ecosystems (Matson, Lohse, & Hall, 2002). For example, plant community structure will alter under the increasing N deposition (Bobbink, Hornung, & Roelofs, 1998; Bragazza et al., 2004; Sala et al., 2000), which is generally associated with the loss in plant species richness and biodiversity (Bobbink et al., 2010; Stevens et al., 2010). As the strong coupling of plant production and N cycles, excess N deposition could significantly impact on ecosystem primary production (LeBauer & Treseder, 2008; Liu & Greaver, 2010; Lu et al., 2011). Additionally, some soil processes, especially the soil N cycle, can be altered by N enrichment (Lu et al., 2011).

Generally, plant biomass is thought to be colimited by water and N availability in natural grasslands (LeBauer & Treseder, 2008; Schenk & Jackson, 2002; St Clair et al., 2009). Although external N input generally increases net ecosystem aboveground primary production by alleviating N limitation (LeBauer & Treseder, 2008; Xia & Wan, 2008), the responses to N changes were also dependent on the amount of N input, rainfall amount, and grassland types, with a greater response in high precipitation regions. Besides the effects on plant biomass, N‐driven reductions in plant species diversity have been reported frequently (Bobbink et al., 1998; Stevens, Dise, Mountford, & Gowing, 2004), which have potentially important implications for both nature conservation and ecosystem functioning (Wamelink et al., 2009). N enrichment can decrease plant species richness by shifting plant communities into the compositions that are able to acquire and/or tolerate high N levels (Bowman, Cleveland, Halada, Hresko, & Baron, 2008). Such losses in plant species diversity, in turn, will influence the ecosystem productivity and stability (Bai, Han, Wu, Chen, & Li, 2004; Tilman, Reich, & Knops, 2006; Tilman, Wedin, & Knops, 1996). A meta‐analysis on N enrichment experiments found greater plant species loss in cold regions and larger increases in plant biomass (Clark & Tilman, 2008). These studies show that besides community composition, the responses of different ecosystems to N enrichment also depend on the climatic factors. Therefore, plant growth and biomass allocation should be predicted under the global climate change. However, there is still no report on how alpine grasslands under different water conditions respond to N addition on the Qinghai–Tibet Plateau (QTP).

N saturation, defined by Aber, Nadelhoffer, Steudler, and Melillo (1989), is the point when the availability of soil ammonium and nitrate exceeds ecosystem N demand. During this process, plant production shifts from an N‐limited, N intermediate, to N saturation stages with the increasing of N addition (Song et al., 2017). The indicators of N saturation have been documented by soil‐based indicators, including the changes in N mineralization, nitrification, and ${\text{NO}}_{3}^{-}$‐N leaching rates (Aber et al., 1989; Bowman, Gartner, Holland, & Wiedermann, 2006; Lovett & Goodale, 2011; Pardo et al., 2011), or the changes in sensitive biota, such as plant community composition (Bowman et al., 2006; Bowman, Murgel, Blett, & Porter, 2012; Lovett & Goodale, 2011). Hence, multilevel N addition experiments are important tools for the estimation of N saturation threshold of terrestrial ecosystems (Bai et al., 2010; Bowman et al., 2006, 2012). According to the N saturation hypothesis, plant production is assumed to firstly increase with N addition rates, then reach the maximum value at the N saturation point, and finally decline with further N input (Aber et al., 1989; Lovett & Goodale, 2011). Previous studies showed that soil properties also responded nonlinearly to the multilevel N additions (Song et al., 2017; Wei et al., 2013). The N saturation threshold of a temperate grassland in Inner Mongolia was 105 kg N hm^−2^ year^−1^ (Bai et al., 2010) and in a semiarid grassland was 91.7 kg N hm^−2^ year^−1^ (Chen, Zhang, Mai, & Shen, 2016). However, the estimation of saturation threshold to N addition rarely carried out in alpine ecosystems on the QTP, which prevents us from accurately predicting alpine grasslands in response to rising N deposition. Alpine grasslands under different climate and nutrient conditions may respond differently to multilevel N addition compared with other grassland types.

The QTP, known as “the Earth's third pole,” is the birthplace of several great rivers in East, Southeast, and South Asian regions (Sun, Zheng, Yao, & Zhang, 2012; Zheng, Zhang, & Yang, 1979). The alpine ecosystems are very fragile and sensitive to global climate change, which could act as “indicator regions” of environmental changes (Tang, Li, & Zhang, 1986). The atmospheric N deposition in remote region was relatively low, ranging from 0.44 kg hm^−2^ year^−1^ in Ngari to 0.92 kg hm^−2^ year^−1^ in Nam Co (Liu, Xu, Wang, Pan, & Piao, 2015); however, it has been increasing in the last several decades dramatically (Jia et al., 2014). In addition, the N deposition rate was relatively high on the east part of QTP, ranging from 8.7 to 13.8 kg N hm^−2^ year^−1^ (Lü & Tian, 2007). However, estimations of N saturation thresholds in response to increasing N deposition have not been reported in alpine ecosystems along the transect on the QTP. Because of the thin soils and low biological buffering capacity, alpine ecosystems are particularly susceptible to continuous N deposition (Bowman et al., 2006; Williams, Baron, Caine, Sommerfeld, & Sanford, 1996). If N input reaches and/or exceeds the saturation threshold, the water resources and ecological security of Asian will be endangered. On the North Tibetan Plateau, the precipitation decreases gradually from east to west. Similarly, soil organic matter content decreases gradually along the precipitation gradient (Zhao et al., 2017). Four types of alpine grassland ecosystems, that is, alpine meadow, alpine meadow‐steppe, alpine steppe, and alpine desert‐steppe, were widely distributed from east to west. Here, we used multilevel N addition experiments in four alpine grasslands to determine the saturation thresholds in the heartland of the QTP; more specifically, we investigated the controlling factors for the saturation thresholds to N addition along the precipitation transect on the QTP.

## 2 MATERIALS AND METHODS

### 2.1 Study site description

The Changtang Plateau (29°53′–36°32′N, 78°41′–92°16′E) is the main part of Tibetan Plateau, located in northwest of Tibetan Autonomous Region, China, with an average altitude of 4,400 m. A remarkable precipitation gradient (<100–700 mm) spans appropriately 1,500 km with successive grasslands of alpine desert, steppe, and meadow from west to east. The alpine vegetation is dominated by alpine steppe with widespread species of *Stipa purpurea* Griseb. and *Carex moorcroftii* Falc. Ex Boott, and a variety of forbs (Wu et al., 2014). N‐fixed legumes, for example, species of *Oxytropis* are widely distributed in western arid part of the Plateau. Soil nutrient is relatively low, with soil organic matter increasing from <1.0% to 4.0% and total N from 0.02% to 0.2%, respectively, from west to east (Zhao et al., 2017). The Plateau is characterized by a cold, arid, and windy climate with sparse and vulnerable vegetation. The general evaporation strength is more than 1,800 mm, with annual mean wind speed more than 3 m/s, and annual mean aridity index ranging from 1.6 to 20 (Mao, Lu, Zheng, & Zhang, 2006). It is extremely cold on the Plateau with mean annual temperature (MAT) <0°C and annual temperature in the warmest month (July) of <14°C in most of the areas (Yang, Zhang, Miao, & Wei, 2003; Zhao et al., 2017). The longitudinal change in MAT is <2°C despite the large precipitation variations along 32°N sampling transect on the Changtang Plateau (Figure S1).

### 2.2 Experimental design and treatment

**Figure 1:**
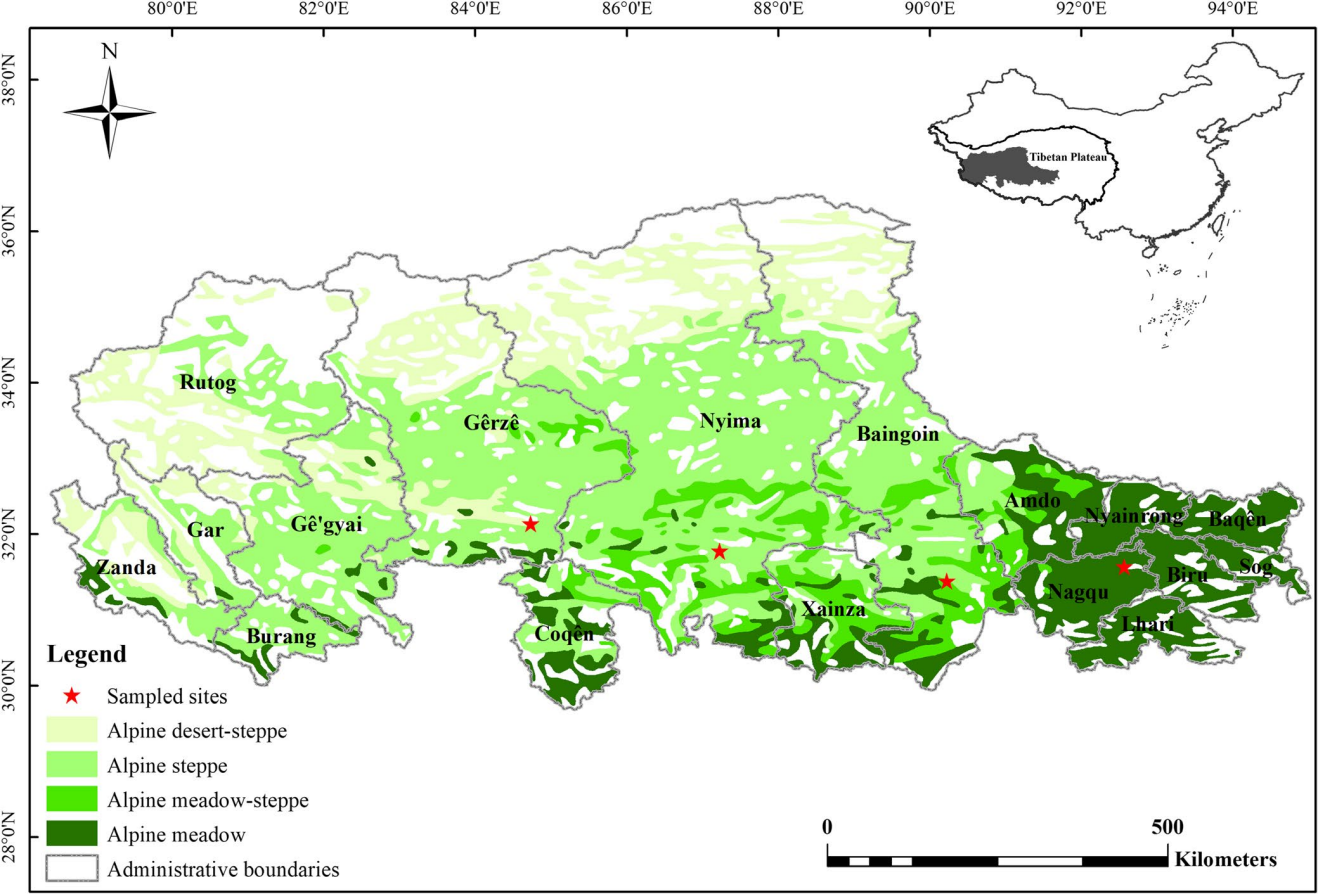
Location of four study sites in alpine meadow (AM) in Nagqu, alpine meadow‐steppe (AMS) in Bangoin, alpine steppe (AS) in Nyima, and alpine desert‐steppe (ADS) in Gerze, respectively

Four alpine grasslands were selected to carry out the fertilization experiments along the precipitation gradient from east to west, that is, alpine meadow (AM), alpine meadow‐steppe (AMS), alpine steppe (AS), and alpine desert‐steppe (ADS) located in Nagqu, Bangor, Nyima, and Gerze, respectively (Figure 1, Table 1). An area of 60 m × 60 m alpine grasslands with uniform vegetation coverage in each study site was selected as the field fertilization experiments. Twenty‐five 4 m × 4 m split plots were laid out in a complete randomized block, designed with five replicates for each of the five treatments which included a control (i.e., N0), and four levels of N enrichment (25, 50, 100 and 200 kg N hm^−2^ year^−1^, hereafter coded as N25, N50, N100, and N200, respectively). Plots were separated by 2‐meter aisles as buffering zones. Granular CO(NH_2_)_2_ fertilizer was directly applied before plant green‐up in each year since 2013 (in mid‐June). All the fertilization plots in these four study sites located in non‐grazing rangelands and excluded from livestock grazing throughout the year.

**Table 1:** Description of the study sites

	Study site			
	AM in Nagqu	AMS in Bangoin	AS in Nyima	ADS in Gerze
Locations	31°34′N	31°23′N	31°47′N	32°22′N
	92°34′E	90°14′E	87°14′E	82°16′E
Altitude (m)	4,570	4,590	4,580	4,520
MAP (mm)	444.9	335.4	327.4	175.2
MAT (°C)	−0.9	−1.0	−1.4	−1.4
Vegetation types	Alpine meadow	Alpine meadow‐steppe	Alpine steppe	Alpine desert‐steppe
Dominated species	*Kobresia pygmaea*	*Stipa purpurea*	*S. purpurea*	*S. purpurea*, *Oxytropis microphylla*

### 2.3 Measurement of plant community and soil properties

Plant community composition within the plots was measured yearly in every August from 2014 to 2017. The mean coverage of each plant species was measured during the peak growing season, which occurs in mid‐August, using a 50 cm × 50 cm quadrat divided into twenty‐five 10 cm × 10 cm subsquares in each plot (Zong et al., 2016). Species richness and the Shannon–Wiener diversity index (*H*′), which represent both the richness and evenness of plant species, were calculated annually in each sampling plot.

To examine the response of the aboveground production of each plant functional group to the N addition treatments, aboveground biomass was clipped in 50 cm × 50 cm subsquares after field surveying within each sampling plot. Biomass was clipped annually during the peak growing season in mid‐August. All the plants were sorted into four functional groups, including grasses, sedges, legumes, and all other forbs. After harvesting, the biomass of each functional group was oven‐dried at 65°C for 48 hr and weighed. The dry matter weight was measured (±0.01 g) and calculated on a per square meter basis. Soil samples (0–20 cm depth) were collected from each soil profiles after the collection of the plant aboveground materials in each growing season. Soil samples were immediately passed through a 2‐mm sieve to remove roots, gravel, and stones and stored below 4°C. ${\text{NO}}_{3}^{-}$‐N and ${\text{NH}}_{4}^{+}$‐N as N content in the composite soil sample were extracted using 2.0 mol/L KCl, filtered, and then analyzed by a continuous flow analyzer (AA3, SEAL Analytical).

### 2.4 Data calculation and statistical analysis

The response function of the change in vegetation coverage or aboveground biomass per year versus the N addition rates was estimated using a polynomial response curve, with the peak values providing the N saturation threshold for vegetation change (Bowman et al., 2006; Zong et al., 2016). We estimated the N saturation thresholds for vegetation coverage or biomass changes as the N deposition rate below or above which community coverage or biomass would decrease (Bowman et al., 2006; Zong et al., 2016).

The vegetation community data (plant species richness, diversity index, community coverage, and aboveground biomass) were analyzed using two‐way (N addition levels and experimental years) analysis of variance (ANOVA) followed by the check of normality. One‐way ANOVA followed by Tukey's multiple comparisons was used to examine the effects of N addition on plant species richness, diversity index, community coverage, and aboveground biomass of total community and each functional group (total biomass, grasses, and legumes) in every sampling year. Regression analysis was used to evaluate the change in community biomass in grasses along with N addition gradients in different sampling years, and the average N saturation thresholds in different grasslands were obtained based on the annual calculation. One‐way ANOVA followed by Tukey's multiple comparisons was also used to examine the effects of N addition on soil inorganic N and total N content in different alpine grasslands. Correlation analysis was also used to analyze the relationships between rainfall and N saturation thresholds as well as between N saturation thresholds and soil inorganic and total N content. The significance level was *p* < .05. All the analyses were performed in SPSS 16.0 (SPSS for Windows, version 16.0), and all the figures were produced by Origin Pro 8.0 (OriginLab Corporation).

## 3 RESULTS

### 3.1 Effects of N addition on plant species diversity

**Figure 2:**
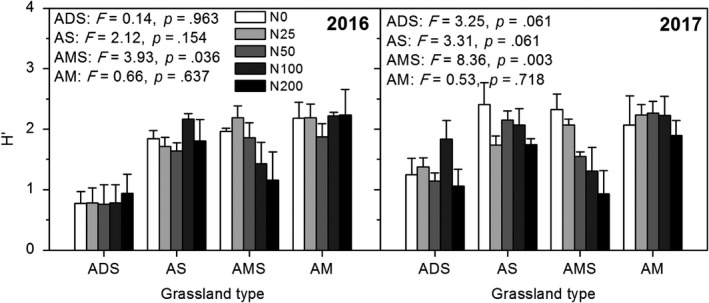
Shannon–Wiener diversity index (*H*′) in alpine meadow (AM) in NQ, alpine meadow‐steppe (AMS) in BG, alpine steppe (AS) in NM, and alpine desert‐steppe (ADS) in GZ in 2016 and 2017, respectively

Both in 2016 and 2017, N addition had a significant impact on plant species diversity in alpine meadow‐steppe (Figure 2, Table 2, *p* < .001). With the increase in N addition rate, plant species diversity showed a decreasing trend. While in alpine meadow, alpine steppe, and alpine desert‐steppe, N addition had no significant effects on plant species diversity, which indicates that the transition zone from alpine meadow to steppe is more responsive to N addition than other alpine grassland types.

**Table 2:** Two‐way ANOVA analysis on plant species diversity (*H*′), and aboveground biomass of total community, grasses, legume biomass in alpine meadow (AM) in Nagqu, alpine meadow‐steppe (AMS) in Bangoin, alpine steppe (AS) in Nyima and alpine desert‐steppe (ADS) in Gerze, respectively, on the Northern Tibetan Plateau, which were subjected to 5 years of ambient deposition (control), and additions of 25, 50, 100, and 200 kg N ha^−1^ year^−1^

Factors	AM in Nagqu						AMS in Bangoin			
	Richness	*H*′	Total	Grass	Legume	Richness	*H*′	Total	Grass	Legume
Year	1.06	0.05	1.94	1.35	0.31	0.60	0.52	30.77***	1.05	0.56
N	1.37	0.32	2.55	0.33	0.92	2.87*	13.59***	1.97	0.50	0.33
Year × N	1.74	0.89	2.08*	27.34***	4.23***	1.09	0.82	1.90*	7.27***	7.03***

Factors	AS in Nyima						ADS in Gerze			
	Richness	*H*′	Total	Grass	Legume	Richness	*H*′	Total	Grass	Legume
Year	10.82**	1.64	24.46***	1.66	0.68	6.84**	11.68	8.48**	2.09	0.34
N	2.83*	1.33	1.55	0.53	0.11	2.71*	0.66	2.32	0.27	0.48
Year × N	1.51	2.36	1.81	4.01***	8.12***	1.47	1.73	2.99**	4.08***	9.20***

* *p* < .05.

** *p* < .01.

*** *p* < .001.

### 3.2 Effects of N addition on community aboveground biomass

**Figure 3:**
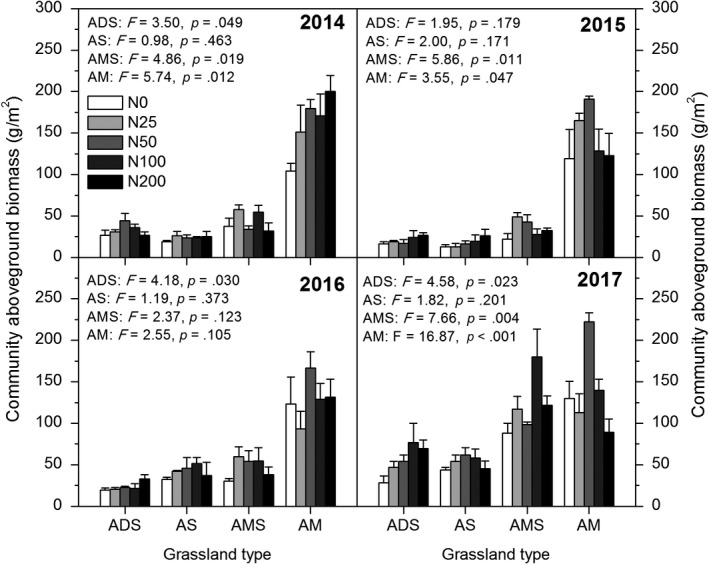
Total community aboveground biomass in alpine meadow (AM), alpine meadow‐steppe (AMS), alpine steppe (AS), and alpine desert‐steppe (ADS) from 2014 to 2017, respectively

Different types of alpine grasslands showed different responses to N addition (Figure 3). The aboveground biomass of desert steppe increased gradually with the increase in N addition rate in 2016 and 2017, but had no significant effects on alpine steppe. Both alpine meadow‐steppe and alpine meadow showed unimodal trends. The mean aboveground biomass of total community also showed a unimodal trend in alpine meadow during 2014 to 2017, while N additions had no significant effect on the other three alpine grassland types (Figure S3).

**Figure 4:**
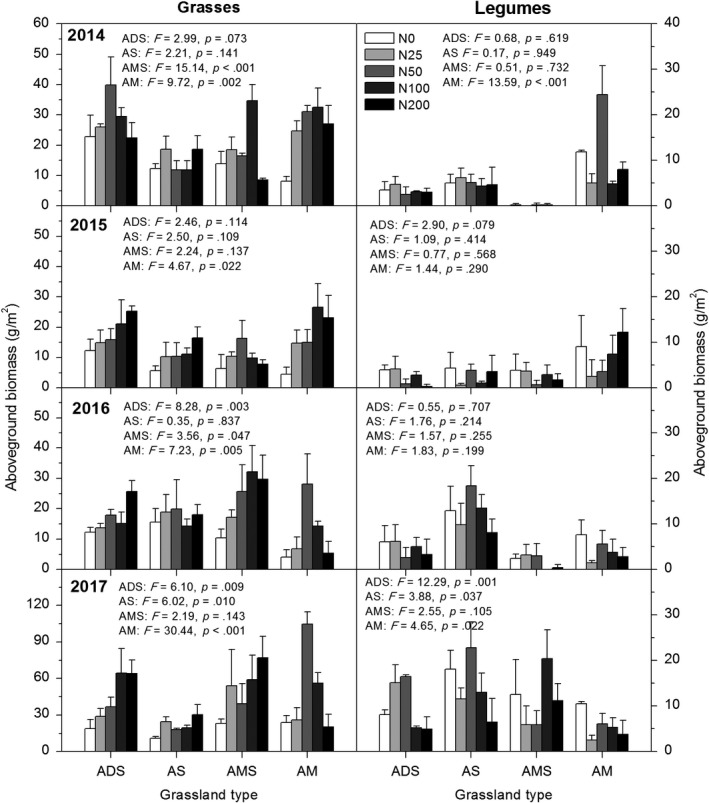
Aboveground biomass of grasses and legume plants in alpine meadow (AM), alpine meadow‐steppe (AMS), alpine steppe (AS), and alpine desert‐steppe (ADS) from 2014 to 2017, respectively

Grasses in different alpine grasslands also responded differently to N addition (Figure 4, Figure S3). With the increase in N addition rate, aboveground biomass of grasses of alpine desert‐steppe showed a gradual increase trend, while in alpine meadow showed a unimodal trend (Figure 4, Figure S3). There was no significant effect on alpine steppe in the first 3 years, and aboveground biomass of grasses showed an increasing trend in 2017 (Figure 4, *p* = .010). In the first 2 years, the aboveground biomass of grasses of alpine meadow‐steppe first increased and then decreased, and then gradually increased in the following 2 years. Aboveground biomass of grasses of alpine meadow showed unimodal trends throughout all the growing seasons.

The mean aboveground biomass of legume plants showed that N addition only had significant effects in alpine meadow during 2014 and 2017 (Figure S3), but they responded differently among years (Figure 4). In the first 3 years, N addition had no significant effect on the legume biomass, except alpine meadow in 2014. In the fifth year of fertilization (2017), N addition had a significant effect on legumes biomass in alpine desert‐steppe, alpine steppe and alpine meadow, while they responded differently to N addition. In alpine desert‐steppe and alpine steppe, legume biomass first increased and then decreased, reaching peak values in N50 (Figure 4). While in alpine meadow, legume biomass showed a decreasing tendency with N addition increase in 2017 (Figure 4, *p* = .022).

### 3.3 Estimation of the N saturation thresholds

**Figure 5:**
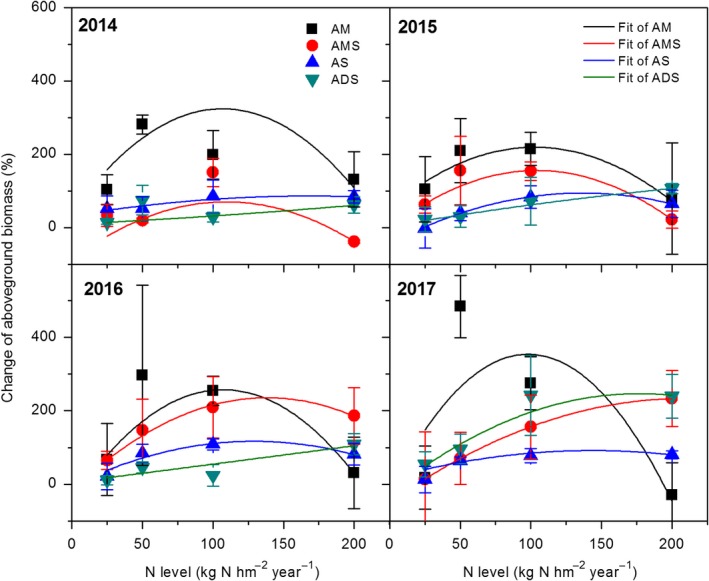
The change in aboveground biomass (data in each year were averaged) for grasses in different alpine grasslands on the Northern Tibetan Plateau subjected to ambient deposition (control), and additions of 25, 50, 100, and 200 kg N hm^−2^ year^−1^. The data were fit with polynomial dose response curves

Total community biomass did not show significant changes, and the most significant change occurred in grasses (Figure 5), so we used the change in grasses to estimate N saturation thresholds. With the increase in N addition rate, the biomass of grasses showed unimodal trends and regression fitting showed a parabolic tendency in grass biomass changes in each sampling years (Figure 5). Based on the correlation analysis in different years, the N saturation thresholds were 103, 115, 136, and 156 kg N hm^−2^ year^−1^ in alpine meadow, alpine meadow‐steppe, alpine steppe, and alpine desert‐steppe, respectively (Table 3). The N saturation threshold in alpine meadow was significantly lower than those in alpine steppe and alpine desert‐steppe (Table 3, *p* = .001). This indicates that alpine grasslands become more and more insensitive to exogenous N input with precipitation decrease.

**Table 3:** The estimation of N saturation thresholds in different alpine grasslands under N treatments in different years

	AM	AMS	AS	ADS
2014	106	109	—	—
2015	102	104	136	155
2016	107	138	129	139
2017	98	109	143	176
Saturation threshold (kg N)	103 (3.5)	115 (13.4)	136 (5.7)	156 (15.2)

Note

The data in the last row represent the average N saturation thresholds in different alpine grasslands, and the data in the brackets represent the standard error of the N saturation thresholds. N saturation thresholds in the last row had significant differences between grassland types (*F* = 12.106, *p* = .001). “—” means no significant correlation and cannot fit to calculate the N saturation thresholds.

The peak values of the fitted parabola represent the maximum promotion of N input on different types of alpine grasslands. The results showed that N input had the greatest promotive effects on alpine meadow (Figure 5). The maximum promotion of biomass to N addition in alpine meadow, alpine meadow‐steppe, alpine steppe, and alpine desert‐steppe was 391.0%, 144.3%, 91.6%, and 116.9%, respectively (Figure 5).

### 3.4 Regulating factors of N saturation thresholds

**Figure 6:**
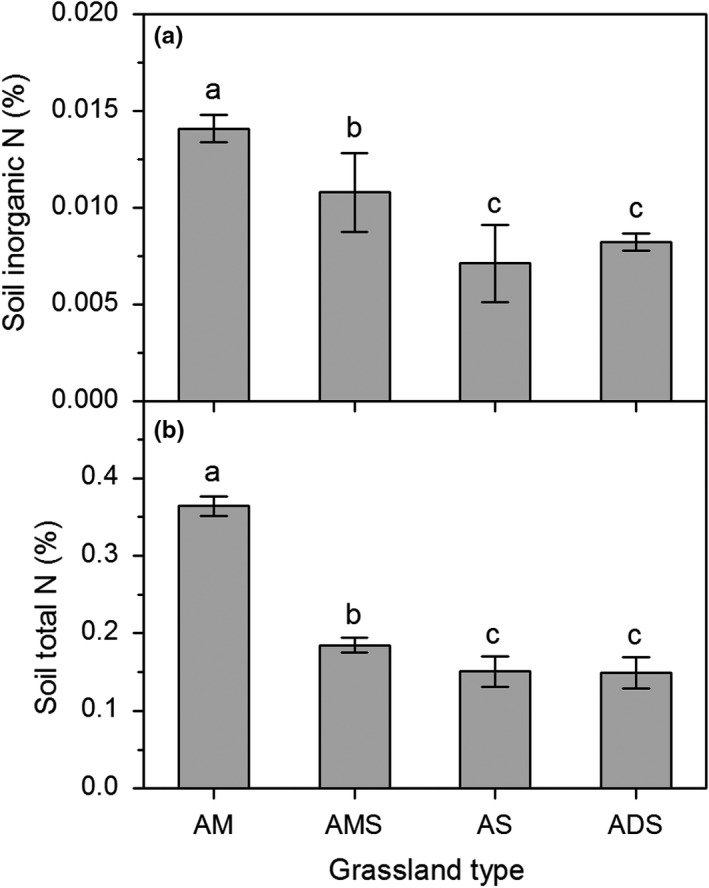
Soil inorganic N (A) and total N content (B) in different alpine grasslands. Different lowercase letters represent the significant difference among different alpine grasslands

The results showed that both soil inorganic N (SIN) and total N content (STN) showed decreasing trends from east to west along the rainfall gradient. SIN and STN in alpine meadow were significantly higher than other grassland types, and SIN and STN in alpine steppe and alpine desert‐steppe showed no significant differences (Figure 6).

**Figure 7:**
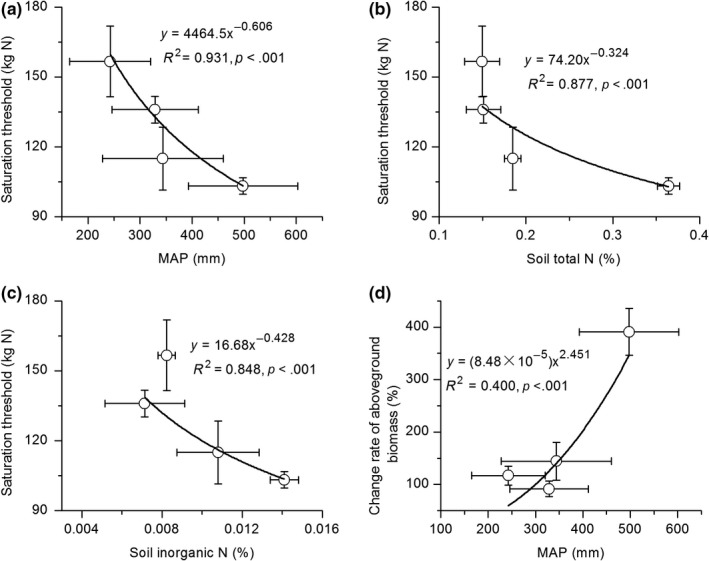
The correlation analysis between mean annual precipitation (MAP, 2013–2017) and N saturation thresholds (A) and maximum promotion of grass biomass (D), as well as the correlation analysis between N saturation thresholds and soil total N (B) and inorganic N content (C)

Along the transect from east to west, the N saturation thresholds of different alpine grassland types gradually increased with the decrease in rainfall, indicating that alpine grasslands are more and more insensitive to increasing drought (Figure 7A). The N saturation thresholds also negatively correlated with STN and SIN (Figure 7B,C). The maximum promotive effect of N input on alpine grasslands showed that it increased exponentially with the increase in rainfall (Figure 7D), indicating the regulating effect of rainfall on exogenous N input in alpine grasslands.

## 4 DISCUSSION

### 4.1 Effects of N addition on different grassland types

In this study, N addition had no effects on plant species diversity in alpine meadow, alpine steppe, and alpine desert‐steppe. Our result is in contrast to most previous studies that showed a loss of plant species richness in response to N addition (Bai et al., 2010; Chen, Lan, Bai, Grace, & Bai, 2013). However, N addition significantly decreased plant species diversity in alpine meadow‐steppe in 2016 and 2017 (Figure 2, Table 2), indicating that the transition zone from meadow to steppe is more responsive to N addition, caused by the strong edge effects and spatial heterogeneity. In addition, the intense ecological processes and complex plant diversity and community structure also give rise to the high sensitivity in these transition zones to global change. This also verified in the changes of plant species richness (Figure S2). In addition to effects on biomass, N‐driven reductions in plant species diversity are frequently reported (Bobbink et al., 1998; Stevens et al., 2004), which has potentially important implications for both nature conservation and ecosystem functioning (Wamelink et al., 2009). A meta‐analysis of N enrichment experiments found greater plant species loss in the communities with cold regional temperatures and larger increases in plant biomass (Clark & Tilman, 2008).

The results showed that the responses of different types of alpine grasslands differed to N addition (Figures 3 and 4, Figures S4 and S5). These different responses may be attributed to the environmental conditions (soil nutrient content and soil water condition), as well as the plant community composition. In our study, the most pronounced functional group showing coverage and biomass increases was grasses, which is similar to the results of other nutrient addition experiments in alpine meadows (Bowman & Conant, 1994; Theodose & Bowman, 1997). Generally, rhizomatous grasses are better adapted to high N levels (Bai et al., 2010). As a faster‐growing and tall species, grasses occupy the upper part of the community canopy and outcompete other species for light (Hautier, Niklaus, & Hector, 2009). Furthermore, the fibrous root systems of grasses have greater abilities to compete for soil water and nutrient resources (Yang, Ren, Zhou, & He, 2014). Recent studies reported that the absorptive capacities of soil organic N and nitrate in the grasses *Poa pratensis* and *S. aliena* were greater than other plant species in an alpine community, as shown by an in situ ^15^N isotope labeling technique (Wang et al., 2012). As a kind of palatable forages, the increase in grasses is beneficial to the recovery of degraded rangelands and the development of livestock husbandry.

On the other hand, *K. pygmaea* is a dominant species in alpine meadow. As is known to all, sedge plants are mainly distributed in the semihumid regions, while the aboveground biomass of sedge plants accounts for a very little proportion to total community (Figure S4). With dense cluster roots, sedge plants generally respond differently to N addition compared with other plant species. Studies in Northwestern Caucasus reported that the response of sedge plants to N addition was positive and much more intensive than other functional groups (Onipchenko et al., 2012), while other studies showed different results. Previous studies in Ellesmere Island showed that sedge plants responded positively only to N+P fertilizer, but did not show any response to N addition only (Henry, Freedman, & Svoboda, 1986; Zong et al., 2014). While other studies in Niwot Ridge, Colorado, demonstrated that N addition could induce a decrease in sedge plants (Bowman, Theodose, Schardt, & Conant, 1993), which is partly consistent with our result (Figure S4). These divergent responses of sedge plants to N addition may be attributed to the differences in climatic conditions and soil nutrient properties, because nutrient additions had different effects in physiognomically similar communities with different water conditions (dry and wet alpine meadows; Bowman et al., 1993). In this study, N addition could enhance the production of sedge plants in the second year of fertilization, while N addition had no effect on sedge plants with N addition year, which indicates that the production of sedge plants is not only limited by N availability, and long‐term higher N addition may have inhibiting effects on sedge plants in these semiarid alpine grasslands.

In the first 3 years, N addition had no significant effect on the legume biomass, except that N fertilization had significant effects on legume plants in alpine meadow in 2014. In addition, legumes biomass responded differently to N addition in different types of alpine grasslands (Figure 4). High N addition rate could inhibit the growth of legumes, which is consistent with other studies (Song et al., 2012). Legume plants are possibly less dependent on soil N availability due to their ability to fix N_2_ in symbiotic association with rhizobia (Yang, Qiao, Xu, & Ouyang, 2011). High N level may inhibit biological N fixation, whereas low N level can promote plant growth and stimulate the formation of rhizobia nodules.

### 4.2 The N saturation thresholds of different grassland types

Grasses were the most sensitive functional groups to exogenous N input, and the changes in grasses biomass showed a parabolic tendency with N increase. N saturation thresholds were 103, 115, 1,336, and 156 kg N hm^−2^ year^−1^ in alpine meadow, alpine steppe‐meadow, alpine steppe, and alpine desert‐steppe, respectively, which indicates that alpine grasslands become more and more insensitive to exogenous N input along with the precipitation decrease. The effects of N addition on alpine grasslands depend on grassland types. At present, the widely accepted conclusion of such studies is that there is an N saturation threshold in ecosystem production (Aber et al., 1989; Bai et al., 2010; Bowman et al., 2006; Chen et al., 2016; Song et al., 2017), that is, low N input can increase aboveground biomass, while high rate can reduce the increase in biomass. Studies have shown that the N saturation threshold of temperate grasslands in Inner Mongolia was 105 kg N hm^−2^ year^−1^ (Bai et al., 2010), and semiarid grassland was 91.7 kg N hm^−2^ year^−1^ (Chen et al., 2016), whereas the N saturation thresholds of alpine grasslands in the Rocky Mountain region of the United States was 46 kg N hm^−2^ year^−1^ (Bowman et al., 2006, 2012), and a semihumid alpine steppe‐meadow on the Tibetan Plateau was 50 kg N hm^−2^ year^−1^ (Zong et al., 2016), which was lower than the results of our study. Generally, alpine grasslands are more sensitive to N enrichment than other types of grasslands due to the higher latitude and low temperature. However, these alpine grasslands in our study are located in arid regions, and the restriction of soil moisture can cause alpine ecosystems to be insensitive to nutrient addition, which is consistent with the negative correlation between precipitation and N saturation thresholds along the precipitation transect. This can also explain the difference between the results in this paper and in semihumid alpine steppe‐meadow. The climate in the semihumid alpine steppe‐meadow is wetter than the alpine grasslands (especially alpine meadow‐steppe, alpine steppe, and alpine desert‐steppe) in this study, which can be explained by the negative correlation between precipitation and N saturation thresholds. In addition, the difference in community composition is also another important cause (Bowman et al., 1993). With higher species richness, both grasses and sedge plants are dominant species in alpine steppe‐meadow, which is more responsive to N addition than the alpine grasslands with relative rare species.

As mentioned above, the sensitivity of alpine ecosystems becomes lower, and soil N content also decreases with precipitation decrease from east to west. Therefore, N saturation thresholds negatively correlated with soil inorganic N and total N content. Generally, N enrichment increased aboveground biomass. However, the response to N was also dependent on the amount of N input and rainfall, and climate conditions can regulate the effects of N addition on alpine grasslands. Previous studies suggested that the sites with high precipitation showed greater aboveground biomass in response to N addition, which means that ecosystems in wet areas may respond more sensitively to N addition. This also reflects the fact that alpine grasslands in these regions (such as the alpine meadow in this study) are more N, and less water limited than those of dry regions (such as alpine steppe and alpine desert‐steppe), and more sensitive to N addition. Low water availability in these dry regions may also prevent the added N from becoming available, as nutrients are not in solution, and alpine grasslands in these regions are insensitive to N addition. In addition, soil microbial activities are generally restricted by soil water content in arid regions, and the nutrient transformation rates are relatively slow, which limits the supply of nutrients to plant growth. Therefore, plant growth in natural grasslands is generally thought to be colimited by water and N availability (Hooper & Johnson, 1999; LeBauer & Treseder, 2008; Schenk & Jackson, 2002; St Clair et al., 2009). Therefore, if the global climate change drivers alter the strength of water and nutrient limitation, there may be large impacts on plant growth and biomass allocation.

### 4.3 The implication of N saturation thresholds estimation

Due to the high latitude, alpine ecosystems on the QTP are especially sensitive to global climate change. In addition, alpine ecosystems are particularly susceptible to N deposition because of their barren soils and low biological buffering capacity (Bowman et al., 2006; Williams et al., 1996). Therefore, if the N input amount reaches and/or exceeds the saturation thresholds in different alpine ecosystems, the ecological security on the QTP will be endangered (Zong et al., 2016). Till now, no similar study has been conducted to estimate the saturation thresholds of different alpine ecosystems in these regions, and long‐term and multiple levels of N input experiments are needed to evaluate the response of vegetation changes on the QTP. The N saturation thresholds identified in this study could be used to predict the response of different alpine ecosystems under atmospheric N deposition increase in future.

In addition, our results indicated that the N saturation thresholds based on the changes in grasses were 103, 115, 136, and 156 kg N hm^−2^ year^−1^ in alpine meadow, alpine steppe‐meadow, alpine steppe, and alpine desert‐steppe, respectively. This estimation is also of great importance for grassland restoration and management. If N addition levels are higher than these N saturation thresholds, they could not yield more forages for livestock, in contrast, leading to even more economic input as well as negative environmental impacts. Sustained, long‐term N addition higher than the N saturation threshold could decrease soil pH, and soil acidification could induce the release of mobile forms of Al^3+^, which are toxic to vegetation in grassland ecosystems (Bowman et al., 2008). Furthermore, a meta‐analysis revealed that long‐term higher N addition would inhibit soil microbial activities, as indicated by a reduction in soil microbial carbon (Janssens et al., 2010; Liu & Greaver, 2010; Treseder, 2008). This result could also provide useful scientific guidelines for the sustainable management of alpine ecosystems on the QTP. In addition, the results showed that alpine grasslands become more and more insensitive to exogenous N input as precipitation decrease. The reduction in N sensitivity caused by the differences in soil moisture and nutrient availability indicates that different alpine grassland types will respond differently to the future scenario of atmospheric N deposition. This different sensitivity of alpine grasslands should be taken into consideration when using fertilization to restore degraded grasslands on the QTP.

## CONFLICT OF INTEREST

The authors declare that they have no conflict of interest.

## AUTHOR CONTRIBUTIONS

NZ and PLS designed the experiments. NZ and GSZ performed the experiments. NZ and GSZ analyzed the data. NZ wrote the manuscript. NZ and PLS revised the manuscript. All authors read and approved the final manuscript.

## Data Availability

Data are available from the Dryad Digital Repository: https://doi.org/10.5061/dryad.8fs1505.
