# Supplementary material for: Different sensitivity and threshold in response to nitrogen addition in four alpine grasslands along a precipitation transect on the Northern Tibetan Plateau
Source: Ecol Evol. 2019 Aug 1;9(17):9782–93. doi: 10.1002/ece3.5514 (PMC6745826; doi:10.1002/ece3.5514)
Supplement: Supplementary file 1 [file ECE3-9-9782-s001.docx]

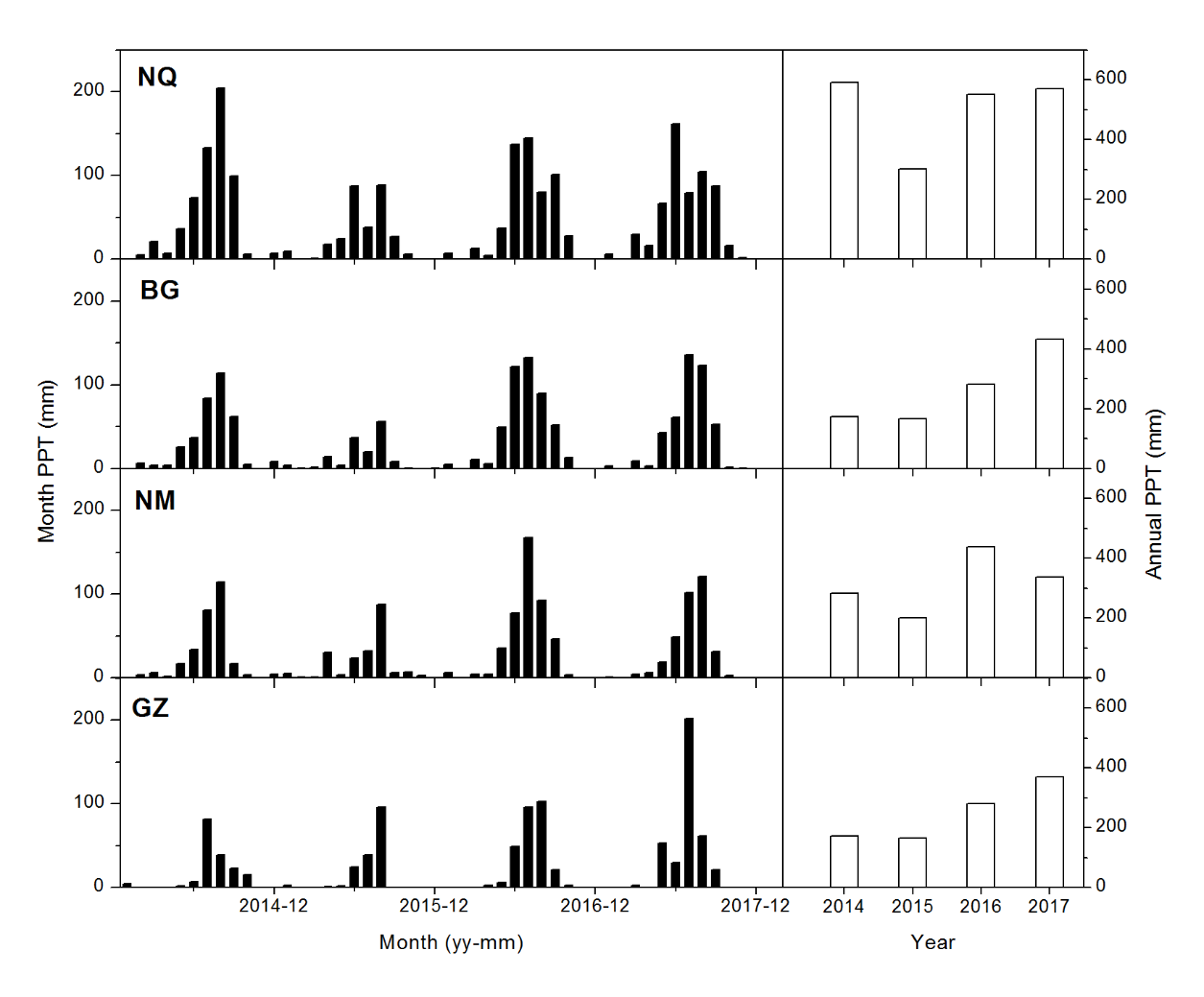


**FIGURE S1** Month and annual precipitation (PPT) in Gerze (GZ), Nyima (NM), Bangor (BG) and Nagqu (NQ) from 2013 to 2017, respectively.


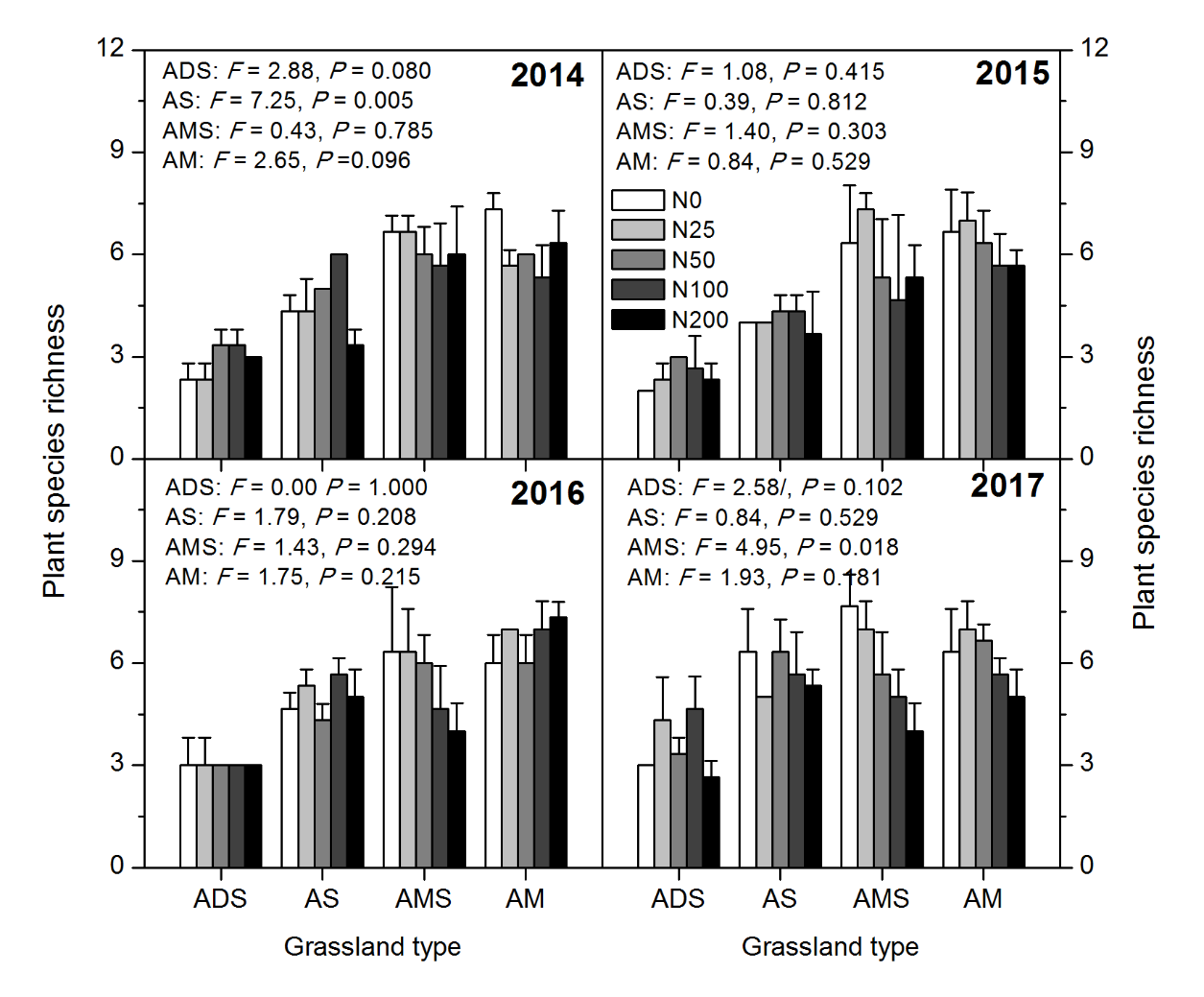


**FIGURE S2** Plant species richness in alpine meadow (AM) in NQ, alpine meadow-steppe (AMS) in BG, alpine steppe (AS) in NM, and alpine desert steppe (ADS) in GZ from 2014 to 2017, respectively.


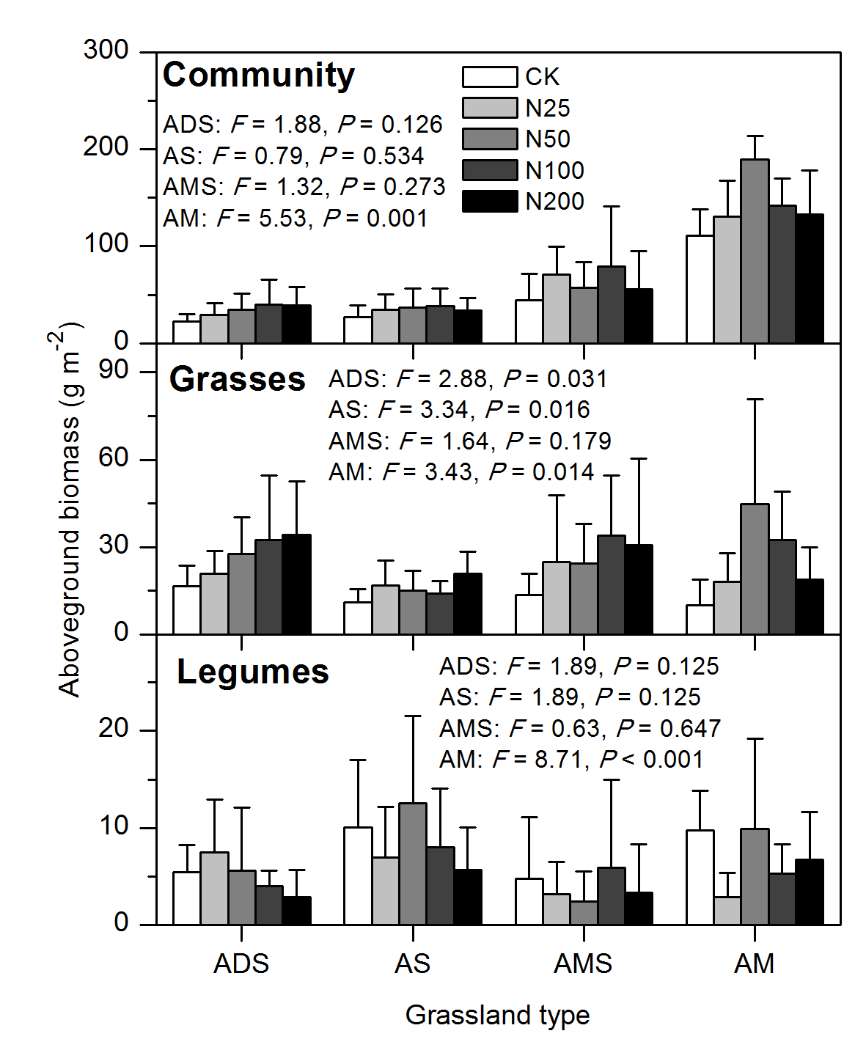


**FIGURE S3** The mean aboveground biomass of total community, grasses and legumes in alpine meadow (AM) in NQ, alpine meadow-steppe (AMS) in BG, alpine steppe (AS) in NM, and alpine desert steppe (ADS) in GZ from 2014 to 2017, respectively.

**
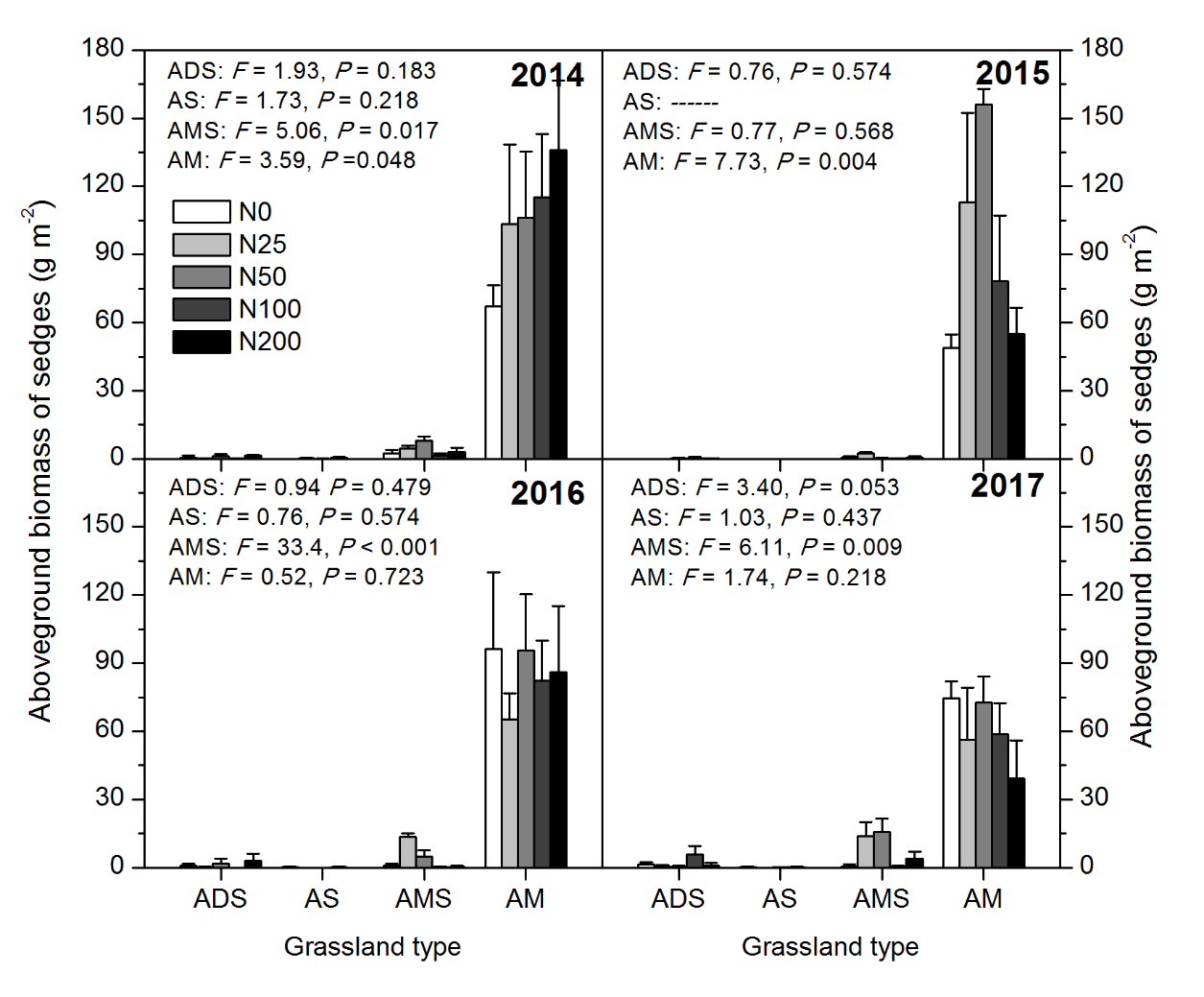
**

**FIGURE S4** The aboveground biomass of sedges in alpine meadow (AM) in NQ, alpine meadow-steppe (AMS) in BG, alpine steppe (AS) in NM, and alpine desert steppe (ADS) in GZ from 2014 to 2017, respectively. NM had no data in 2015, so data analysis did not be conducted in this year.


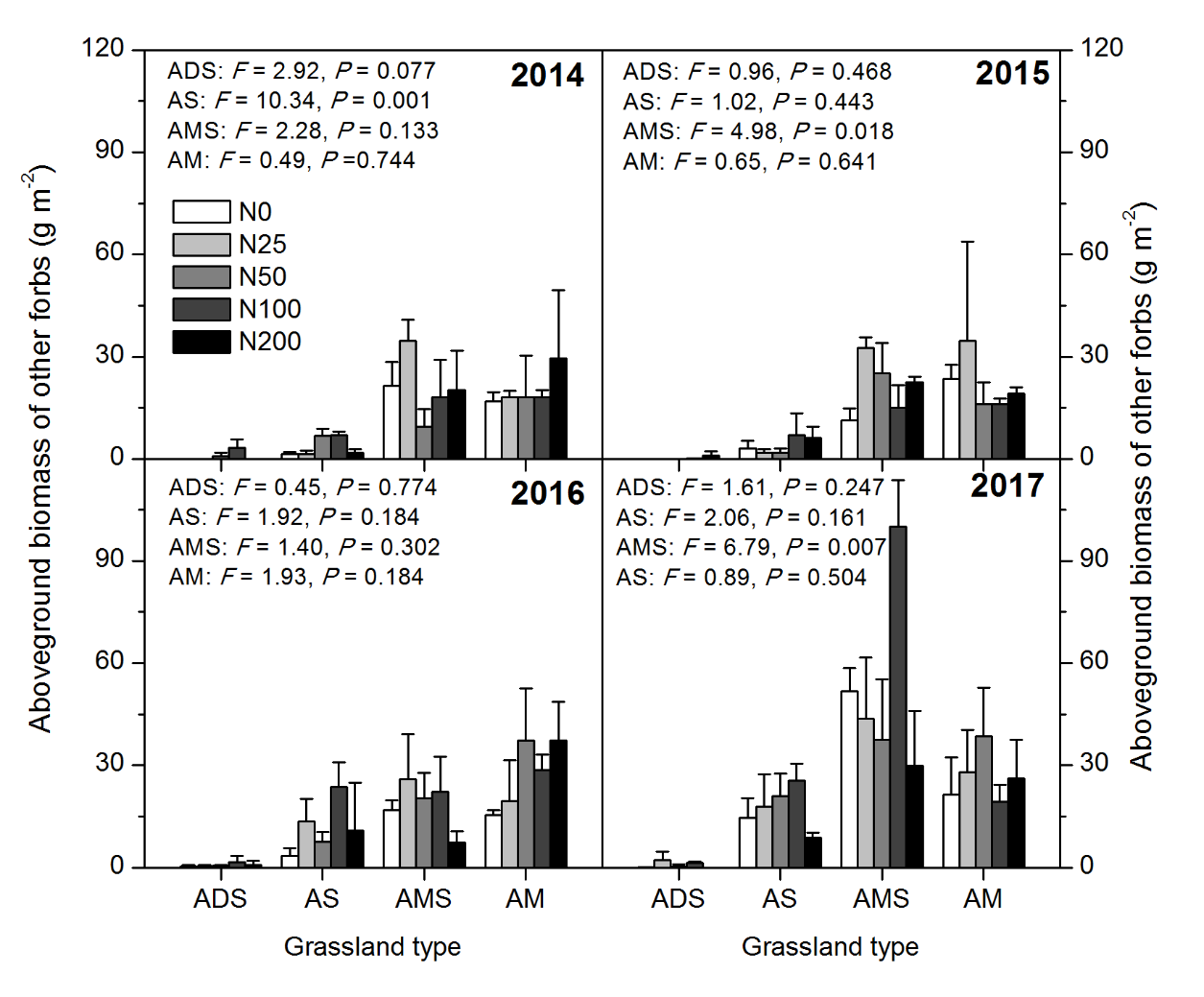


**FIGURE S5** The aboveground biomass of other forbs in alpine meadow (AM) in NQ, alpine meadow-steppe (AMS) in BG, alpine steppe (AS) in NM, and alpine desert steppe (ADS) in GZ from 2014 to 2017, respectively.
